# Complex Shapes Are Bluish, Darker, and More Saturated; Shape-Color Correspondence in 3D Object Perception

**DOI:** 10.3389/fpsyg.2022.854574

**Published:** 2022-05-04

**Authors:** Jiwon Song, Haeji Shin, Minsun Park, Seungmin Nam, Chai-Youn Kim

**Affiliations:** School of Psychology, Korea University, Seoul, South Korea

**Keywords:** intra-modal correspondence, cross-modal correspondence, shape, color, visuo-haptic, 3D shape

## Abstract

It has been shown that there is a non-random association between shape and color. However, the results of previous studies on the shape-color correspondence did not converge. To address the issue, we focused on shape complexity among a number of shape properties, particularly in terms of 3D shape, and parametrically manipulated the shape complexity and all three components of color. With two experiments, the current study aimed to closely examine the correspondence between shape complexity of 3D shape and color in terms of hue (Experiment 1), luminance, and saturation (Experiment 2). Participants were presented with the 3D shapes in either visual or visuo-haptic modes of exploration. Subsequently, they had to pick from a color palette the color best matching each shape of the object. In Experiment 1, we found that as shapes became more complex, the best associated hue changed from those with long wavelengths to ones with short wavelengths. Results of Experiment 2 demonstrated that as the shapes grew more complex, the associated luminance decreased, and saturation increased. Additionally, adding haptic exploration to visual exploration strengthened the association – for saturation in particular – with the pattern of shape-color correspondence maintained. Taken together, we demonstrated that complex shapes are associated with bluish, darker and more saturated colors, suggesting that shape complexity has a systematic relationship with color including hue, luminance, and saturation.

## Introduction

Our brain tends to associate a certain feature in one sensory modality with a feature in another modality in a non-random manner, which is known as “cross-modal correspondence” (Marks, 2004; Spence, 2011). For example, auditory pitch is known to be associated with visual luminance (Wicker, 1968; Marks, 1974), shape (Marks, 1987; Walker et al., 2010), and size (Gallace and Spence, 2006; Parise and Spence, 2009; Evans and Treisman, 2010; Bien et al., 2012). In addition, tactile properties such as vibrotactile frequency/amplitude and haptic properties such as smoothness, softness, and roundness are systematically related to visual features such as lightness and color (Martino and Marks, 2000; Ludwig and Simner, 2013; Slobodenyuk et al., 2015; Delazio et al., 2017). Such correspondence between perceptual features occurs not only across sensory modalities but also within a single modality. In recent years, there has been a growing interest in this intra-modal correspondence, especially within the visual modality between numerous visual properties (size, direction, amount, intensity; Lindauer, 1969) or graphemes (Simner et al., 2005; Lau et al., 2011) and color. One of the most representative types of intra-modal correspondence is shape-color correspondence.

A classic shape-color correspondence was first suggested by Kandinsky in the early 20th century. He proposed a fundamental association between primary shapes and colors (triangle with yellow, square with red, and circle with blue) based on the survey he conducted (Droste, 1990; Lupton and Miller, 1991; Gage, 1993). However, subsequent surveys attempting to replicate Kandinsky’s results did not converge (Jacobsen, 2002; Jacobsen and Wolsdorff, 2007; Kharkhurin, 2012). Also, studies utilizing the Implicit Association Task (IAT) failed to verify the associations postulated between Kandinsky’s three shapes and colors though they hypothesized that participants would show faster RTs when the response key mapping matched the proposed associations (Makin and Wuerger, 2013; Chen et al., 2015b).

Previous studies have mainly utilized discrete primary geometric shapes (i.e., circle, triangle, square, and hexagon) and colors (i.e., red, yellow, and blue) in a one-to-one matching task (Makin and Wuerger, 2013; Chen et al., 2015b,^2016^). However, such cases have not considered that shapes have multiple properties such as complexity, pointedness, symmetry, and regularity, each of which is influential of the outcome shape (Pizlo, 2010). Among a few studies of shape-color correspondence that have acknowledged this, shape complexity has been a topic of interest (Malfatti, 2014; Chen et al., 2016; Dreksler and Spence, 2019). Several studies manipulated shape complexity into multiple levels (Malfatti, 2014; Dreksler and Spence, 2019). Specifically, in Malfatti (2014), shape complexity was considered by the number of points into three levels but was not found to be associated with color (luminance and saturation). In a subsequent study by Dreksler and Spence (2019), the extent of shape complexity was changed into three or four levels with respect to the number of sides or levels of concavities. However, inconsistent results were found in the different experiments of the study: complex shape showed a relationship with both dark (Experiments 1 and 2) and light colors (Experiment 3).

Next, color is composed of three elements: hue, luminance, and saturation. In contrast to most previous studies which only included hue in the discourse of shape-color correspondence, recent research began considering all three elements when investigating whether a specific shape is associated with a specific color. For example, in the study of Dreksler and Spence (2019), ten hues were chosen and presented at two different levels of luminance (light and dark) and at full saturation. Malfatti (2014) employed a color set consisting of eight hues, provided at two levels of luminance (light and dark) and saturation (saturated and muted). However, the results of the relationship between shape complexity and luminance/saturation of these two studies were inconsistent; a close association between complexity and luminance was shown in one study (Dreksler and Spence, 2019), whereas complexity was not associated with luminance and saturation in the other (Malfatti, 2014). Commonly, both studies divided luminance and saturation into two levels, though luminance and saturation vary continuously along each dimension. In the present study, to account for the continuity of shape and color features, we parametrically manipulated levels of shape and color. Specifically, we generated shapes whose complexity was manipulated in three levels, and colors in which their three elements (hue, luminance, and saturation) were shown on a continuous scale. By using these stimuli, we expected to gain a deeper understanding of shape-color correspondence.

A notable addition of the current study is the realistic 3D objects used as stimuli. Previous imaging studies have suggested overlapping activations in the brain during visual and haptic processing of 3D objects (Amedi et al., 2001; Kim and James, 2010). That there is a transfer of object shape information between vision and haptics was also supported by behavioral results (Gibson, 1963; Newell et al., 2001; Norman et al., 2004). Taken together, shape properties are extracted from objects not only by visual exploration but also haptic exploration of those objects, and exploring objects in both visual and haptic modalities would provide a more comprehensive percept of 3D shapes. By using 3D shape stimuli and exploring the object both visually and haptically, we expected the added haptic modality to facilitate the effects of visual shape-color correspondence.

With two experiments, the current study aimed to closely examine the patterns of correspondence between shape complexity and color in terms of hue (Experiment 1), luminance, and saturation (Experiment 2). In Experiments 1 and 2, participants were presented with the 3D shapes – in either visual (video clips of 3D objects) or visuo-haptic (viewing 3D-printed objects while exploring it with their hands) modes of exploration. Subsequently, they had to pick from a color palette the color best matching each shape of the object. To examine the systematic patterns of correspondence between shape complexity and color, we parametrically manipulated the levels of both shape and color stimuli. The shapes of 3D objects were manipulated into three levels of complexity. For the color stimuli, hue was presented at eight levels (Experiment 1) and both luminance and saturation were divided into fifteen levels (Experiment 2). In addition, utilizing 3D shapes allowed us to test the modulatory effect of exploration modality. Reflecting the way our stimuli were manipulated, we hypothesized that shape complexity is systematically related to color including hue, luminance, and saturation. We also hypothesized that the non-arbitrary mapping between shape complexity and color is modulated by the modality of exploration (i.e., visual or visuo-haptic).

## Materials and Methods

### Participants

One hundred ninety participants (58 males and 132 females, 20–35 years of age) participated in Experiment 1, which was an online experiment. Twenty-seven participants (14 males and 13 females, 20–32 years of age) took part in Experiment 2. All participants had normal or corrected-to-normal visual acuity and normal color vision. They gave informed consent approved by the Korea University Institutional Review Board (KUIRB-2020-0174-01).

### Apparatus

Apparatus differed for participants in Experiment 1 because it was an online experiment and individuals had freedom to choose their own device. For Experiment 2, visual stimuli were presented on a 19-inch CRT monitor (HP v930, 1024 × 768 resolution, 60 Hz refresh rate, viewing distance 55 cm). Haptic stimuli were created by printing out the 3D shape models using Stratasys’ J750. The experiment was conducted in a quiet, dark room using MATLAB version 9.5 (The Mathworks, Inc., Natick, MA, United States) and Psychophysics Toolbox version 3 (Brainard, 1997; Pelli, 1997).

### Stimuli

#### Shape

**FIGURE 1:**
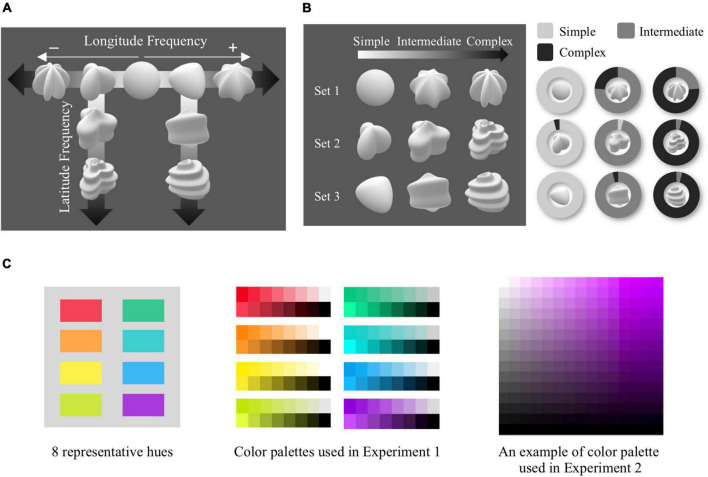
Shape model, conditions, and color palettes. **(A)** Nine shape models manipulated in complexity were created by modulating longitude and latitude frequencies in Superformula. **(B)** Three complexity sets used in Experiments 1 and 2 are shown in the left panel. The right panel shows the results of a survey to confirm whether the manipulated complexity levels were in line with the perceived shape complexity. The ratio of each shape ranked as simple/intermediate/complex is shown as the area within the pie chart. **(C)** Eight representative hues used in experiments are shown in the left panel. From those hues, eight color palettes in the form of brief overview of luminance and saturation were used in Experiment 1 (center panel). An example of the color palette that consists of luminance and saturation dimensions is shown in the right panel, which was used in Experiment 2.

The 3D shape stimuli consisted of nine achromatic shapes created with a variant of Superformula (Gielis, 2003). Superformula was simulated by using an interactive WebGL app.^1^ Luminance and saturation values were identical across all shapes. To manipulate the complexity of the shape, we first generated a sphere shape–starting from this sphere, we created shapes of which complexity was parametrically modulated by increasing longitudinal frequency in both positive and negative values of a parameter. The increased frequency resulted in an increased number of sides either inflated (positive value) or deflated (negative value) on the 3D shapes, which was the basic characteristic defining complexity in our stimuli. Thus, an increased number of sides indicated more complex shapes. Out of the generated shapes, we selected the two three-edged shapes and created four more shapes by increasing latitudinal frequency from either shape. Thus, a total of nine shape models were created (Figure 1A).

The three shapes in each column display complexity controlled in three levels–simple, intermediate, and complex. As shown in the left panel of Figure 1B, the two different columns each constitute a shape set (see Supplementary Videos 2, 3), and the remaining three shapes in the row constitute the last shape set (see Supplementary Video 1). The three complexity sets are contingent upon the base shape from which shape complexity was manipulated. Through an additional online survey (*N* = 25), we attempted to confirm whether each set’s complexity levels manipulated in latitude or longitude were in line with perceived shape complexity. Within each of the complexity sets, participants rated perceived complexity by listing the shapes from simplest to most complex. We found that the perceived complexity of participants mirrored the manipulated complexity across all shape sets, confirming that shape complexity was successfully manipulated in the shape models (right panel of Figure 1B).

For Experiment 1 and the visual condition in Experiment 2, video clips panning the stimuli from various angles were created for each of the shape models in order to give participants a complete view of the three-dimensional shape stimuli. Haptic stimuli for the visuo-haptic condition in Experiment 2 were printed out with the *x*-axis fixed to 60 mm so participants could explore them freely in their hands. They all had a smooth white surface to match that of the visual stimuli.

#### Color

As shown in the left panel of Figure 1C, we selected eight hues, consisting of four approximately unique hues (red, green, yellow, blue) and their approximate angle bisectors (orange, chartreuse, cyan, purple), adopted from Palmer and Schloss (2010). In Experiment 1, we created a color palette for each of the eight hues. The color with a specific hue can be expressed not in a single color, but in various colors depending on the luminance and saturation values. To provide a diverse range of colors that an identical hue can have, we produced a palette consisting of 2 × 8 color chips with eight variants of luminance and saturation, respectively. In Experiment 2, a palette consisting of luminance and saturation axes was created for each hue. Luminance and saturation dimensions of the palette were parametrically manipulated in HSV color space by 15 × 15 color chips, mapped onto a rectangle (right panel of Figure 1C). There were two versions of the palette–upright and inverted–which were randomized across trials.

### Procedures

#### Experiment 1

**FIGURE 2:**
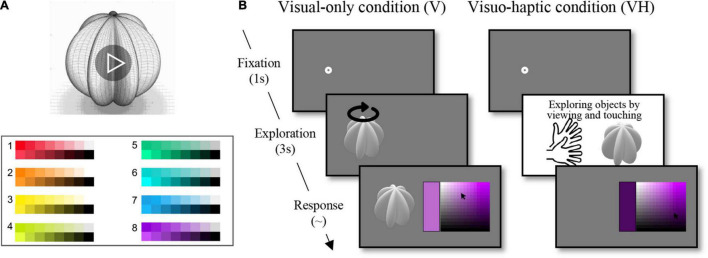
Procedures in Experiments 1 and 2. **(A)** Participants watched each of the nine video clips panning an achromatic 3D shape for 3 s and chose from eight hue palettes which hue they thought best matched the shape. **(B)** 1s fixation was followed by 3 s of a video clip in the visual-only condition and by 3 s of viewing and exploring a 3D shape object in the visuo-haptic condition. After the exploration, participants were presented a palette manipulated in luminance and saturation dimensions for a hue and chose the color they thought best matched the shape.

Data were collected online using Google Forms (Mountain view, CA, United States).^2^ There were three different versions of the survey depending on stimulus presentation order, all in which presentation order was pseudo-randomized. Each participant only responded to one of the three versions. Participants watched each of the nine video clips panning an achromatic 3D shape for 3 s, and chose from eight color palettes which they thought best matched the shape each time it was presented (Figure 2A). The video was presented only once for each shape.

#### Experiment 2

The experiment was composed of two blocks depending on the modality of exploration condition (visual-only and visuo-haptic), with the visual-only block preceding the visuo-haptic block. In the visual condition, participants viewed each of the video clips for 3 s, then were presented with a palette manipulated in luminance and saturation dimensions for a hue (Figure 2B and Supplementary Videos 1–3). Using a computer mouse, they indicated the color they thought best matched the shape. By clicking on a part of the palette (i.e., a color chip), they could explore the color in a larger window, and save their response with another click on the window. Based on the results from Experiment 1, the best associated hues for each of the nine shapes were ranked in a descending order. We took five hues that had the best matched, highest percentage of responses for each shape from the eight hues, respectively (see Table 1). Five palettes for each shape were presented once each. With nine shapes, five hues, and two upright/inverted palettes, participants completed 90 trials. The order of trials was randomized.

**TABLE 1:** The five hues that had the best matched, highest percentage of responses for each of the nine shapes.

		1st	2nd	3rd	4th	5th
Simple	Set 1	Red	Blue	Yellow	Cyan	Purple
	Set 2	Orange	Yellow	Green	Chartreuse	Red
	Set 3	Red	Orange	Yellow	Chartreuse	Green
Intermediate	Set 1	Orange	Red	Blue	Yellow	Cyan
	Set 2	Yellow	Green	Orange	Chartreuse	Red
	Set 3	Cyan	Orange	Blue	Chartreuse	Yellow
Complex	Set 1	Orange	Red	Yellow	Purple	Chartreuse
	Set 2	Green	Cyan	Purple	Blue	Red
	Set 3	Blue	Cyan	Purple	Orange	Yellow

In the subsequent visuo-haptic condition, participants were handed a white 3D shape object. They viewed the object while exploring it with both hands for 3 s. After the 3 s of exploration, the object was taken from them. Then the participants were presented with a palette and responded in the same manner as the visual condition (Figure 2B). Again, each haptic 3D shape object was presented five times, each time with a different color palette as in the visual condition. The order in which the palettes were presented was randomized. Participants completed 90 trials for the visuo-haptic condition as well.

## Results

### Experiment 1

#### Shape Complexity in Relation With Hue

We investigated the hue that is best associated with the shape according to the shape complexity level. We calculated the percentage of each hue selected for each shape for each complexity set. We then organized the shapes based on their complexity levels (i.e., simple, intermediate, complex) and averaged the calculated percentage for hue.

**FIGURE 3:**
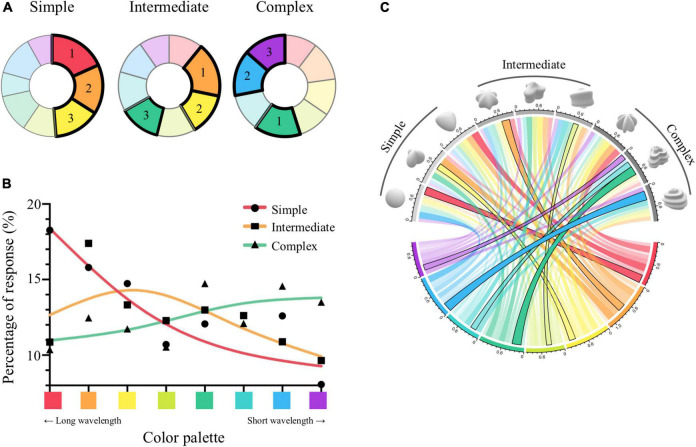
Results from Experiment 1. **(A)** Pie charts illustrate the percentage of responses for each hue according to complexity condition, indicated by the area on the chart for each color. Hues ranked as the first, second, and third are marked with bold lines and numbers. **(B)** Distributions of the percentage of responses for hues were fitted depending on the complexity level using spline interpolation. The *x*-axis indicates the eight color palettes. *Y*-axis is the percentage of the hue choice for complexity condition (simple, intermediate, complex). **(C)** The chord diagram illustrates the degree of association between each shape and color palette. Shapes are presented in order of complexity condition on the upper half circle. Colors on the lower half circle indicate the eight hues. Color transparency of the connected line shows the percentage of responses. Black outlines of the links indicate the connection of a shape with the highest % response in each hue.

As shown in the pie chart of Figure 3A, we found that percentage of responses for each hue changed depending on shape complexity, and that, as the shapes became more complex, the best matches changed gradually from hues with long wavelength (i.e., red, orange, yellow) to ones with short wavelength (i.e., green, blue, purple). To assess whether there is a relationship between shape complexity and hue, we conducted a Chi-Square test of independence. Results showed that distributions of the percentage for hues vary depending on complexity (χ^2^ = 45.291, *p* < 0.001). *Post-hoc* paired comparisons showed that hue distribution of simple condition was statistically significantly different from those of intermediate and complex conditions (simple-intermediate, χ^2^ = 20.555, *p* = 0.012; simple-complex, χ^2^ = 32.183, *p* < 0.001, intermediate-complex, χ^2^ = 13.427, *p* = 0.186, Bonferroni corrected). Similarly, when distributions of the percentage for hues were fitted depending on the complexity level using spline interpolation (Figure 3B), the fitted distributions were each biased toward short, medium, and long wavelength hues. We also investigated the degree of associations according to which shape is most associated with each color palette (a chord diagram in Figure 3C). Results showed that complex shapes had stronger connections with short wavelength hues (i.e., green to purple), and the simpler the shape, the stronger the connection with long wavelength hues (i.e., yellowish-green to red). In sum, that the shapes with higher complexity displayed a tendency to be matched with shorter wavelength hues was proven consistently across multiple representations of the results.

### Experiment 2

#### Shape Complexity in Relation With Luminance and Saturation

As an extension of the relationship between shape complexity and hue in Experiment 1, we further explored the associations between the complexity and luminance/saturation and the impact of exploration modality (i.e., visual-only or visuo-haptic) on the associations. Based on the results from Experiment 1, we took five hues that had the best matched, highest percentage of responses for each shape from the eight hues. Participants chose a color chip that they thought would best match the shape in a palette with modulated luminance and saturation. We collected the luminance and saturation values of the chosen color. The two values were averaged, respectively, across all the color palettes within each shape.

**FIGURE 4:**
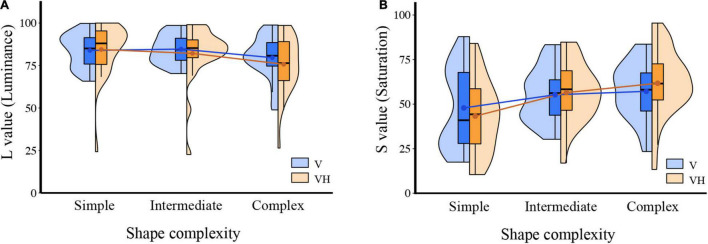
Results from Experiment 2. **(A)** Violin plots illustrate the estimated distributions of individual mean L value (luminance) in each shape complexity (simple, intermediate, complex) and modality exploration (visual-only, V; visuo-haptic, VH) conditions. The middle horizontal lines on the box plots indicate the median, and the lower and upper ends indicate the first and third quartiles. Dots on the box plots indicate the mean. **(B)** Violin plots are shown for the result of S value (saturation).

We conducted two-way repeated-measures ANOVAs with the factors “shape complexity” (i.e., simple, intermediate, complex) and “modality of exploration” (i.e., visual-only, visuo-haptic) for luminance and saturation. Results are shown in Figure 4. As for luminance, the interaction effect between shape complexity and modality of exploration was not statistically significant [*F*_(2, 50)_ = 1.834, *p* = 0.17, $\eta_{p}^{2}$ = 0.068]. The main effect of shape complexity was statistically significant [*F*_(2, 50)_ = 8.892, *p* < 0.001, $\eta_{p}^{2}$ = 0.262], but that of modality of exploration was not significant [*F*_(1, 50)_ = 0.669, *p* = 0.421]. *Post-hoc* Bonferroni comparisons of shape complexity showed that luminance value was lower in the complex level than in the simple or intermediate level [simple-intermediate, *t*(50) = 0.544, *p* = 1; simple-complex, *t*(50) = 3.051, *p* = 0.016, Cohen’s *d* = 0.598; intermediate-complex, *t*(50) = 4.011, *p* = 0.001, Cohen’s *d* = 0.787]. These findings suggest that as the shape grew more complex, it was associated with a color with lower luminance. The pattern of association did not differ depending on what the exploration modality was.

As for saturation, the interaction effect was statistically significant [*F*_(2, 50)_ = 6.432, *p* = 0.003, $\eta_{p}^{2}$ = 0.205]. The main effect of shape complexity was statistically significant [*F*_(2, 50)_ = 14.739, *p* = 0.002, $\eta_{p}^{2}$ = 0.371] but that of exploration modality was not significant [*F*_(1, 50)_ = 0.018, *p* = 0.894]. *Post-hoc* Bonferroni comparisons of shape complexity showed that saturation value was higher in the complex level than in the simple or intermediate level [simple-intermediate, *t*(50) = −3.9, *p* = 0.002, Cohen’s *d* = −0.765; simple-complex, *t*(50) = −3.972, *p* = 0.002, Cohen’s *d* = −0.779; intermediate-complex, *t*(50) = −2.57, *p* = 0.05, Cohen’s *d* = −0.504]. These results suggest that higher saturation values are associated with more complex shapes. In addition, the pattern of association was modulated according to the modality of exploration, meaning that participants showed a more pronounced modulation of responses according to shape complexity in the visuo-haptic condition when compared to the visual condition for saturation while only showing a tendency to do so for luminance.

## Discussion

The present study demonstrates a non-arbitrary association between shape and color. Importantly, using parametrically manipulated 3D shape and color stimuli enabled us to more closely examine the shape-color correspondence. The associated colors showed systematic modulations according to the complexity of 3D shapes. Specifically, results of Experiment 1 showed that as shapes became more complex, the best associated hue changed from those with long wavelengths to ones with short wavelengths. In Experiment 2, we found that with increasing shape complexity, the associated luminance decreased and the associated saturation increased. Moreover, when haptic exploration was combined with visual exploration, there was an added effect to the association between shape complexity and elements of color–for saturation in particular. The results imply a non-random mapping between shape complexity and color including hue, luminance, and saturation. It also suggests the importance of considering haptic characteristics of shape when examining shape-color correspondence.

Our results of Experiment 1 are in line with those from previous studies to show associations between 2D shape complexity and hues. Although results from three experiments of Dreksler and Spence (2019) were not consistent, their second experiment manipulating complexity into four levels demonstrated that shape with low-medium complexity was associated with yellow (Dreksler and Spence, 2019). Chen et al. (2016) examining cross-preference of shapes and colors showed that people who preferred simple shapes tend to prefer yellowish colors while people who preferred complex shapes tend to prefer bluish colors. Additionally, early studies examining shape-color correspondence with primary geometric shapes (i.e., circle, triangle, square, and hexagon) and colors (i.e., red, yellow, and blue) implied that shapes with more angles, namely complex shapes, are associated with hues with short wavelengths (Jacobsen and Wolsdorff, 2007; Albertazzi et al., 2013; Chen et al., 2015a,^2016^).

Our study is distinguished from previous studies by investigating luminance and saturation on a continuum. For instance, Dreksler and Spence (2019) manipulated luminance into two levels and showed conflicting results: associations between complex shape and dark color, and associations between complex shape and light color. In the study of Malfatti (2014), luminance and saturation were manipulated into two levels, respectively. However, the relationship between shape complexity and luminance/saturation was not found. It is probable that existing associations between shape and colors were not revealed because previous studies merely manipulated the stimuli in a binary manner. Dividing the wide spectrum of both luminance and saturation into multiple levels enabled us to examine the relationship between shape complexity and color in a more systematic manner. Therefore, we could find compelling evidence for a non-random mapping between shape complexity and luminance/saturation.

We used 3D modeling to create objects that enable dynamic haptic exploration, which proved to have several advantages. In our everyday lives, we encounter and interact with 3D objects. Among these interactive experiences, shape perception is a multisensory experience not only of vision but also including haptics. This has been evidenced by both behavioral and neuroscientific studies (Gibson, 1963; Amedi et al., 2001; Newell et al., 2001; Ernst and Banks, 2002; Norman et al., 2004; Kim and James, 2010). It is plausible that this multimodal characteristic of shape perception influences shape-color correspondence. Unlike previous studies that have only utilized 2D shapes, we used 3D shape stimuli which can be explored by both visual and haptic modalities to account for the multimodal nature of shapes when examining shape-color correspondence.

Using the 3D stimuli, our results displayed tighter patterns of associations in the multimodal exploration condition. Specifically, the association observed in the visual-only condition was significantly strengthened in the visuo-haptic condition for saturation while showing a tendency to do so for luminance. The results of visuo-haptic exploration are displaying a similar pattern of results with visual exploration can be attributed to the fact that vision and haptics share common brain regions such as the middle occipital (MO) cortex (James et al., 2002), the lateral occipital (LO) cortex (Malach et al., 1995; Grill-Spector et al., 2001), the lateral occipital tactile-visual (LOtv) (Amedi et al., 2001, 2002) and the intraparietal sulcus (IPS) (Culham and Kanwisher, 2001; Grefkes et al., 2002; Zhang et al., 2004; Stilla and Sathian, 2008). These results can also be understood in relation to the origin of cross-modal correspondence. One explanation for cross-modal correspondence is the long-term learning that reflects the statistical landscapes of everyday experience (Parise, 2016). As mentioned earlier, perception in our daily lives is more often multisensory than unisensory. Thus, tighter shape-color associations portrayed in the visuo-haptic exploration condition–which is closer to experience in everyday life–supports the statistical explanations of cross-modal correspondence.

The current study revealed that perceptual inputs from a single modality or different sensory modalities are closely associated with each other in a systematic and non-random manner. Yielding results that serve as significant empirical evidence for intra-modal/cross-modal correspondence between shape and color, the present study entailed several methodological advantages. First, parametrically manipulating the level of shape complexity and color and presenting the wide spectrum of both luminance and saturation allowed us to systematically examine the shape-color correspondence. Second, utilizing 3D shapes enabled us to consider the multimodal nature of shapes when examining shape-color correspondence. Our results have an important implication in the sense that it has potential for application in the industrial field. There have been attempts to apply the knowledge of cross-modal correspondence to branding and marketing (Klink, 2003; Lowrey and Shrum, 2007). In a similar vein, the current results could be a useful source for a design reflecting our innate associations between intra-modal/cross-modal features. Taken together, the present study provides a basic understanding of systematic shape-color correspondences with a possibility for application.

## Data Availability Statement

The raw data supporting the conclusions of this article will be made available by the authors, without undue reservation.

## Ethics Statement

The studies involving human participants were reviewed and approved by the Korea University Institutional Review Board. The patients/participants provided their written informed consent to participate in this study.

## Author Contributions

JS, HS, MP, SN, and C-YK designed the experiment, analyzed the results, and revised the manuscript. JS, HS, MP, and SN conducted the experiment. JS, HS, MP, and C-YK drafted the manuscript. All authors contributed to the article and approved the submitted version.

## Conflict of Interest

The authors declare that the research was conducted in the absence of any commercial or financial relationships that could be construed as a potential conflict of interest.

## Publisher’s Note

All claims expressed in this article are solely those of the authors and do not necessarily represent those of their affiliated organizations, or those of the publisher, the editors and the reviewers. Any product that may be evaluated in this article, or claim that may be made by its manufacturer, is not guaranteed or endorsed by the publisher.

## Footnotes

1
http://andrewmarsh.com/software/supershapes-web/

2
https://forms.gle/N68bVn2YF6tDiswy5

## Supplementary Material

Supplementary Video 1 Shape set 1. Three shapes of set 1 are presented in the order of complexity. They were also used as stimuli in the visual condition of Experiment 2.

Supplementary Video 2 Shape set 2. Three shapes of set 2 are presented in the order of complexity. They were also used as stimuli in the visual condition of Experiment 2.

Supplementary Video 3 Shape set 3. Three shapes of set 3 are presented in the order of complexity. They were also used as stimuli in the visual condition of Experiment 2.
